# Blood Plasma Markers in Depressed Mice under Chronic Social Defeat Stress

**DOI:** 10.3390/biomedicines12071485

**Published:** 2024-07-05

**Authors:** Dmitry A. Smagin, Dmitry V. Bezryadnov, Maria G. Zavialova, Anastasia Yu. Abramova, Sergey S. Pertsov, Natalia N. Kudryavtseva

**Affiliations:** 1 Federal Research Center, Institute of Cytology and Genetics, Siberian Branch, Russian Academy of Sciences, Novosibirsk 630090, Russia; 2 Federal Research Center for Innovator and Emerging Biomedical and Pharmaceutical Technologies, P.K. Anokhin Research Institute of Normal Physiology, Moscow 125315, Russia; bezriadnov@gmail.com (D.V.B.); pertsov_ss@academpharm.ru (S.S.P.); 3 Institute of Biomedical Chemistry, Moscow 119121, Russia; mariag.zavyalova@gmail.com; 4 Pavlov Institute of Physiology, Russian Academy of Sciences, Saint Petersburg 199034, Russia

**Keywords:** chronic social stress, depression, blood plasma proteome, lipid metabolism

## Abstract

It has previously been shown that, in mice, chronic social defeat stress in daily agonistic interactions leads to a depression-like state similar to that in depressive patients. With this model, it has become obvious that it is possible to study peripheral markers of the depression-like state in an experiment. This paper was aimed at searching for protein markers in the blood plasma of depressed mice in the chronic social conflict model, which allows for us to obtain male mice with repeated experiences of defeat. Proteomic analysis of blood plasma samples was conducted to identify proteins differentially expressed in this state. There were changes in the expression levels of the amyloid proteins SAA1, SAA4, and SAMP and apolipoproteins APOC3, APOD, and ADIPO in the blood plasma of depressed mice compared with controls (unstressed mice). Changes in the expression of serine protease inhibitors and/or proteins associated with lipid metabolism, inflammation, or immune function [ITIH4, SPA3, A1AT5, HTP (HP), CO9, and A2MG] were also found. Here, we showed that chronic social stress is accompanied by increased levels of amyloid proteins and apolipoproteins in blood plasma. A similarity was noted between the marker protein expression changes in the depressed mice and those in patients with Alzheimer’s disease. These data indicate a psychopathogenic role of chronic social stress, which can form a predisposition to neurodegenerative and/or psychoemotional disorders.

## 1. Introduction

Major depressive disorder (MDD) is a widespread mental illness. In the development of depression, an important role is played by the interactions between genetic, environmental, or stress factors [1]. Numerous studies are being conducted to find peripheral markers of depression in people; such data will help to devise treatment strategies for this disorder, e.g., [2,3,4,5]. The analysis of differentially expressed proteins in MDD has revealed significant alterations in lipid transport and metabolic function, including some apolipoproteins and a serine protease inhibitor, in patients in comparison with healthy controls [6]. In that work, the top statistically significant pathways were related to lipid transport, inflammation, and immunity. It is also known that, in patients with MDD, severe psychological distress and anxiety are accompanied by the development of amyloidosis ([7,8], review). Moreover, in humans, depression is considered a common comorbidity of Alzheimer’s disease [9,10]. It is important to find peripheral biomarkers of depression in the hope to understand preferential comorbidity of depression with other diseases. This knowledge will help design a strategy for treating the disease and its consequences. Nonetheless, this approach requires preliminary studies in animal models. To this end, it is necessary to conduct preliminary research on animal models that can maximally reproduce the psychoemotional state and somatic changes characteristic of people having a diagnosis of a depressive disorder.

It has been shown earlier in experimental studies that chronic social defeat stress caused by negative social experiences in daily agonistic interactions leads to a depression-like state accompanied by chronic anxiety in male mice [11,12,13,14]. This model has been identified as the most relevant for researching depression and the consequences of chronic social stress [15,16] and is widely used for experimental work [17,18]. This is because this state has been confirmed by all validity criteria proposed for experimental models of depression [19]: the similarity of etiology, symptoms, sensitivity to antidepressants and anxiolytics, and neurochemical changes in the brain similar to those in depressed patients. In various tests after chronic social stress, the defeated mice have demonstrated new types of behavior that are similar to symptoms in depressed patients: a general behavioral deficit in various tests, helplessness, generalized anxiety, immobility, freezing, avoidance of social contacts, decreased sociability, indifference, an anhedonia-like state, weight loss, and long persistence of depression- and anxiety-like states [13,14]. Chronic social stress and anxiety are considered the main psychopathogenic factors.

It has also been shown in experiments that the mechanism of the depression-like state is accompanied by the development of immunosuppression (review, [20]), manifesting itself as a decrease in overall resistance to diseases, impairments of humoral and cellular immunity and of proliferation-related and apoptotic processes in immunocompetent organs, and intensification of oncogenic processes. Primary treatment with anxiolytics and antidepressants to correct psychoemotional status has been found to be more effective in comprehensive reversal of psychoneuroimmune impairments than attempts to restore immune metrics with the aim of producing therapeutic effects on anxious–depressive states and immunity (review, [20]). These data indicate a deep connection between changes in the brain and the peripheral pathophysiology of depression accompanied by immune disorders. This notion has given hope of detection, for example, of the stage and depth of development of psychoemotional disorders using peripheral biomarkers.

The aim of this study was to search for proteins of various peripheral-blood-plasma components in depressed mice using the chronic social conflict model [12,13,14] and, for comparison, in the aggressive mice after such daily agonistic interactions resulting in the development of psychosis-like behavior that is accompanied by hyperactivity, enhanced aggressiveness, aggressive motivation, development of stereotypies, and a disturbance of social recognition [21,22]. A comparison of the peripheral changes in mice with different psychoemotional-like disorders will reveal which changes are specific to which condition.

## 2. Materials and Methods

### 2.1. Animals

Adult C57BL/6 male mice were obtained from the Animal Breeding Facility (Stolbovaya, Moscow region, Russia). The animals were housed under standard conditions (at a constant temperature of 22 ± 2 °C, under a 12:12 h light/dark regimen starting at 8:00 a.m., with food in pellets and water available ad libitum). Mice were weaned at 3 weeks of age and housed in groups of 8–10 in standard plastic cages (36 × 23 × 12 cm). The experiments were performed on 10–12-week-old animals. All procedures were in compliance with the European Communities Council Directive 210/63/EU of 22 September 2010. The protocol of this study was approved by the Scientific Council No. 9 of the Institute of Cytology and Genetics SB RAS of 24 March 2010, decision No. 613 (Novosibirsk, http://spf.bionet.nsc.ru; accessed on 9 October 2023).

### 2.2. The Chronic Social Conflict Model

**Figure 1:**
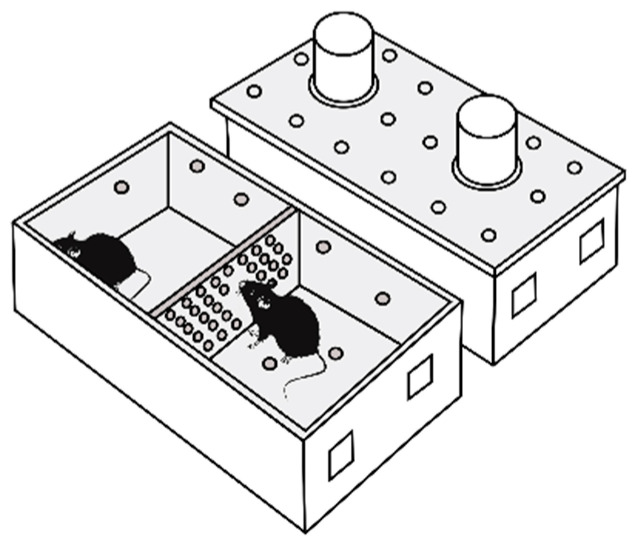
The experimental cage.

Repeated positive and negative social experiences in male mice were induced by daily agonistic interactions through the use of the sensory contact model [21], which was later renamed as the “chronic social conflict model” and is described in detail in some reviews [14,22]. Pairs of male mice were each placed in a cage (28 × 14 × 10 cm) bisected by a transparent perforated partition, allowing the animals to see, hear, and smell each other but preventing physical contact (Figure 1).

The animals were left undisturbed for 2 days to adapt to the new housing conditions and sensory contact before they were subjected to agonistic encounters. After that, every afternoon (3:00–5:00 p.m. local time), the cage cover was replaced by a transparent one, and 5 min later (the period necessary for activation), the partition was removed for 10 min to encourage agonistic interactions. The superiority of one of the mice was established within two or three encounters with the same opponent. The superior mouse would be chasing, biting, and attacking the other, who would be demonstrating only defensive behavior (e.g., upright or sideways postures, withdrawal, and freezing). To prevent damage to the defeated mouse, the aggressive interactions between males were discontinued by lowering the partition if the strong attacking behavior lasted for 3 min (in some cases less). Each defeated mouse (loser) was exposed to the same aggressive mouse for 3 days, whereas afterwards, each loser was placed into an unfamiliar cage containing an unfamiliar winning partner behind the partition. Each aggressive mouse (winner) remained in its own cage. This procedure was performed once a day for 20 days and yielded equal numbers of losers and winners.

In this experiment, three groups of animals were analyzed: (1) controls, i.e., mice without a consecutive experience of agonistic interactions; (2) losers, depressed mice, i.e., a group of repeatedly defeated mice during 20 days of agonistic interactions; (3) winners, i.e., a group of repeatedly aggressive and winning mice during 20 days. The latter group was examined to determine the group specificity of changes in comparison with the losers. The number of mice used for the proteomic analysis was 6 per group. The winners and losers the day after the last agonistic interaction and the control animals were decapitated simultaneously. Blood plasma was collected, frozen, and stored at −80 °C until the proteomic procedures.

### 2.3. The Partition Test

This test is used for studying the level of communicativeness (sociability) toward a partner. The partition test estimates a mouse behavioral reaction to a conspecific behind the transparent perforated partition dividing the experimental cage into equal parts. It has been demonstrated earlier ([23], review) that a decrease in some parameters measured by the partition test correlates with indices of anxiety-like behavior estimated in the elevated plus-maze test in the losers. The behavior of winners near the partition as a reaction to a partner in the neighboring compartment has correlated with parameters of aggression, such as total attacking time, the number of attacks, and average time of one attack, reflecting the level of aggressive motivation [23].

In the current work, the number of approaches to the partition and the total time spent near it (e.g., moving near the partition, smelling and touching it with the nose or with one or two paws, and sticking the nose into the holes) were scored during 5 min as indices of reacting to the partner. The duration of a sideways position or “turning away” near the partition was not included in the total time of the test. On the testing day, the cage lid was replaced with a transparent one; five minutes later (period of activation), the behavioral responses of the losers and winners toward the partner in the adjacent compartment were recorded for 5 min.

### 2.4. Proteomic Data Processing and Analysis

The proteomic analysis was carried out at the Advanced Mass Spectrometry Core Facility of the Institute of Science and Technology (Moscow, Russia). The aim of this study was to compare the protein profile of plasma samples between the groups of mice using label-free quantitative proteomics (LFQ). The plasma samples were trypsin-digested by the manufacturer’s S-trap protocol [S-Trap™ micro use 4.7, ProtiFi LLC, https://files.protifi.com/protocols/; accessed on 20 June 2022]. Eighteen samples of trypsinized mouse blood plasma were analyzed by liquid chromatography coupled with tandem mass spectrometry (LC–MS/MS) similarly to a previously published study [24]. The proteomic analysis was performed by means of the MaxQuant software (ver. 1.6.3.4) using the SwissProt sequence database for *Mus musculus* (mouse). Proteins were identified with the application of criteria defined by the international proteomic organization HUPO (Human Proteome Organization [25]). A total of 249 mouse proteins were found in the samples (Supplement, Table S1). Proteins were identified in at least two technical replicates by means of two or more peptides, whose sequences were confirmed by a tandem mass spectrum.

### 2.5. Functional Annotation of the Proteins

This procedure was performed using the DAVID (Database for Annotation, Visualization, and Integrated Discovery) gene annotation tool [https://david.ncifcrf.gov/; accessed on 25 December 2023]. The *Mus musculus* genome served as the background list for over-representation analysis. The Gene Ontology (GO) option in DAVID as well as the Kyoto Encyclopedia of Genes and Genomes (KEGG) pathway database [https://www.genome.jp/kegg/pathway.html; accessed on 16 October 2023] were used to identify significantly (*p* < 0.05) enriched biological processes and metabolic pathways. An atlas of combinatorial transcriptional regulation in mice and humans was employed to identify differentially expressed genes encoding transcription factor genes [26]. The STRING: functional protein association networks [https://string-db.org/; accessed on 25 December 2023] and GeneCards: The Human Gene Database [https://www.genecards.org; accessed on 25 September 2023] were used to construct a functional protein association network.

### 2.6. Statistical Analysis

Statistical analysis of the data obtained during the protein identification was carried out in the Perseus v.1.6.15.0 software. The data were first filtered to select the most significant points: Possible contaminating and decoy proteins were removed, and proteins that were present in two of three technical replicates of each sample were retained. The plasma samples were grouped according to the behavioral experiences of the mice: “winners, aggressive mice,” “losers, depressed mice,” and the control group of mice. Statistical analysis of the samples based on the LFQ values of the identified proteins made it possible to find patterns in the distribution of proteins in the samples. Proteins were identified in at least two technical replicates by means of two or more peptides whose sequences were confirmed by a tandem mass spectrum. Principal component analysis (PCA) was performed to test the possibility of clustering of the data about the identified proteins of the mouse groups. To detect significant differences between the proteins of the mouse groups, a pairwise comparison of groups by the LFQ protein content was made using a Volcano plot based on the t test with a false discovery rate (FDR) adjustment of Benjamini and Hochberg (with q = 5%). STATISTICA 12.0 (StatSoft, Tulsa, OK, USA), JACOBI4 (software for multivariate analysis of biological data) [w1. JACOBI4: https://jacobi4.ru; accessed on 21 November 2019], and XlStat (xlstat.com; accessed on 16 October 2023) software were employed for the data analysis and presentation. 

## 3. Results

### 3.1. Animal Weight

The defeated mice (losers) and aggressive mice (winners) were weighed before and after the 20 days of agonistic interactions (Table 1). Before the experiments, the weight of the mice did not differ between the groups. After the 20 days of the experiment, the weight of the losers was significantly less than that of the winners.

### 3.2. The Partition Test

**Figure 2:**
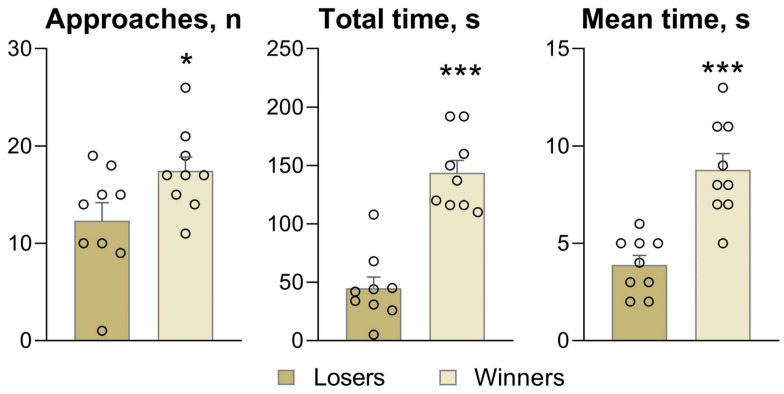
Social reactions of losing and winning mice to a partner in an adjacent compartment of the cage in the partition test. * *p* < 0.05 and *** *p* < 0.001 for a comparison of winners with losers. Statistical significance was set at an FDR-adjusted *p* value < 5%.

Behavioral data in the partition test (Figure 2), as in our previous experiments [23], confirmed that the number of approaches to the partition (as a reaction to the partner in an adjacent compartment) and total and mean time near the partition are significantly less in the depressed losers than in the winners. These results indicated that the losers demonstrated a high level of anxiety (by avoiding the socialization) and low motor activity in comparison with the winners, which exhibited high motor activity and strong aggressive motivation toward the partner in the neighboring compartment.

### 3.3. Analysis of Differentially Expressed Proteins in Blood Plasma of Male Mice

Based on the statistical analysis of the samples in the Perseus v.1.6.15.0 software, 15 proteins were found that were reproducibly present in technical replicates and whose amounts differed significantly between groups of plasma samples (complied according to behavioral experience in the daily agonistic interactions). Furthermore, we paid special attention to the proteins that significantly differed in expression between the groups in the t test corrected for the FDR at *p* < 0.05. Table S2 (Supplement) presents the description of the functions of proteins and genes associated with diseases according to STRING databases for *Mus musculus*.

**Figure 3:**
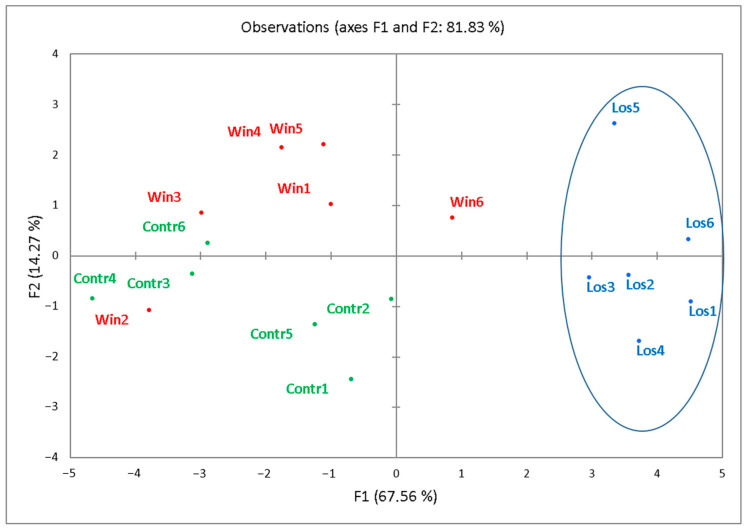
A PCA plot of the distribution of samples according to the average LFQ values of mouse plasma proteins differentially expressed (*q* < 0.05) in at least one of the mouse groups. Green dots denote the control group (Contr), red dots represent the winners (Win), and blue dots represent the losers (Los) in the comparison “Contr–Los,” “Contr–Win,” or “Los–Win.”

Judging by our data (Figure 3), the plasma samples from the losers were separated from the control group and winners. The control and winner groups of samples were not distinguishable by PCA.

**Figure 4:**
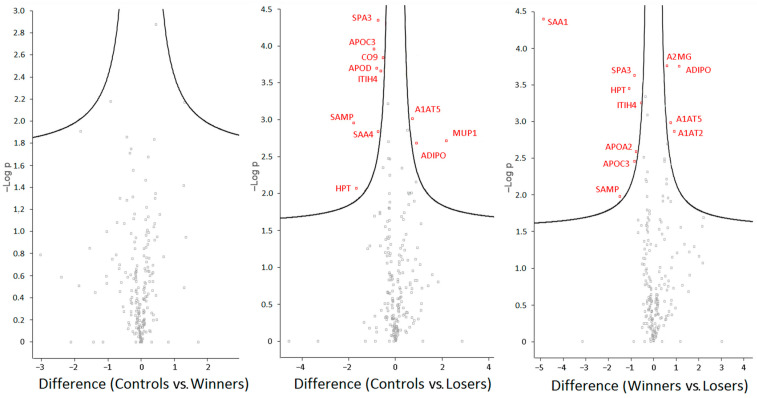
The volcano plot comparing proteins of different groups of mice (Controls vs. Winners, Controls vs. Losers, or Winners vs. Losers). The x-axis is the range of differences; the y-axis is the statistical significance. Red: proteins with statistically significant differences, gray: proteins with statistically insignificant differences.

No significant differences in the protein content were found between the controls and winners. The depressed mice differed from the control group and the winners in 11 proteins (Figure 4). The data showed that, in blood plasma, the main families of proteins that showed an association between the depression-like state and altered expression were amyloid, apolipoproteins, trypsin inhibitors, serine peptidase inhibitors, complement components, and globular proteins.

**Figure 5:**
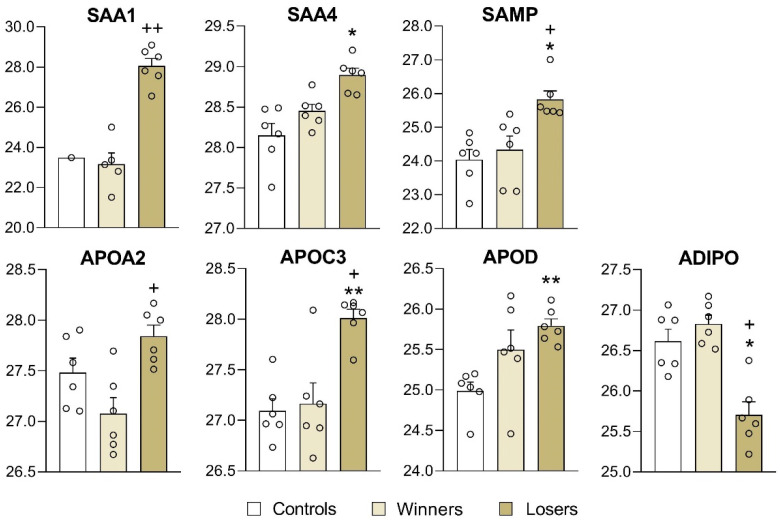
Plasma amyloid proteins and apolipoproteins of interest in the controls, winners, and losers (depressed mice). Controls vs. Losers: * *q* < 0.05; ** *q* < 0.01; and Winners vs. Losers: ^+^ *q* < 0.05; ^++^ *q* < 0.01. Data are presented as log2 LFQ intensity values. Statistical significance was set at an FDR-adjusted *p* value < 5%. All data on differentially expressed proteins are presented in Supplement: Table S3 for the Controls vs. Losers comparison, Table S4 for the Controls vs. Winners, and Table S5 for the Winners vs. Losers.

For a detailed analysis, we selected the proteins that changed expression only according to FDR-adjusted (*q* < 0.05) values. These were amyloid protein SAA4 as well as SAMP: serum amyloid A-4 protein and serum amyloid P-component, encoded by genes *Saa4* and *Apcs*, respectively (Figure 5). The level of the SAA1 protein, encoded by genes *Saa1,* was significantly higher in the depressed mice than in the winners (*p* < 0.000; *q* < 0.0082). The level of the SAA4 protein was significantly higher in the depressed mice in comparison with the control mice (*p* < 0.0015; *q* < 0.0303) or the winners (*p* < 0.0037; *q* < 0.0457). The SAMP protein was significantly overexpressed in the depressed mice in comparison with the control mice (*p* < 0.0011; *q* < 0.0286) and winners (*p* < 0.0105).

Higher levels in the blood plasma of the depressed mice in comparison with the control mice were registered for the proteins APOC3 (*p* < 0.0001; *q* < 0.0089) and APOD (*p* < 0.0002; *q* < 0.0089), and lower levels were registered for ADIPO proteins (*p* < 0.0021; *q* < 0.0333) (apolipoprotein C-III, apolipoprotein D, and adiponectin, encoded by genes *Apoc3*, *Apod*, and *Adipoq*, respectively). The level of APOA2 (apolipoprotein A-II, encoded by the *Apoa2* gene) was higher in comparison with the winners (*p* < 0.0026; *q* < 0.0447) (Figure 5).

**Figure 6:**
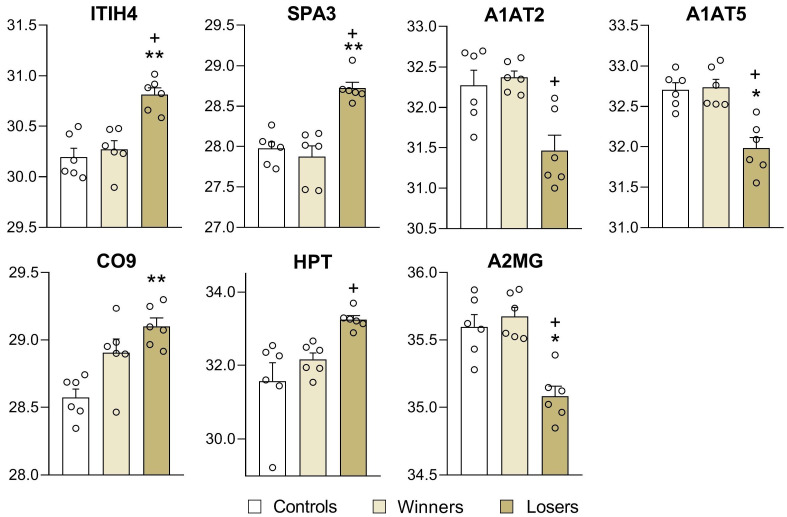
Plasma serine protease inhibitors (proteins) and globulins in the controls, winners, and losers (depressed mice). Controls vs. Losers: * *q* < 0.05; ** *q* < 0.01; Winners vs. Losers: ^+^ *q* < 0.05; Data are presented as log2 LFQ intensity values. Statistical significance was set at an FDR-adjusted *p* value < 5%. All data on differentially expressed proteins are presented in Supplement: Table S3 for the Controls vs. Losers comparison, Table S4 for Controls vs. Winners, and Table S5 for Winners vs. Losers.

The proteins ITIH4, SPA3 (SPA3N, SPA3A, and SPA3G), A1AT2, and A1AT5, which are encoded by genes *Itih4*, *Serpina1b*, *Serpina1e*, and *Serpina3* (*Serpina3n*, *Serpina3A*, and *Serpina3G*), respectively, are broad-spectrum protease inhibitors (Figure 6). Higher levels of these proteins were found in the depressed mice in comparison with the controls for ITIH4 (*p* < 0.0002; *q* < 0.0089), SPA3 (SPA3N) (*p* < 0.0000; *q* < 0.0089), CO9 (*p* < 0.0001; *q* < 0.0089), and HPT (*p* < 0.0087), and lower levels were found for A1AT5 (*p* < 0.0010; *q* < 0.0286), and A2MG (PZP) (*p* < 0.0014; *q* < 0.0303). The “winners vs. losers” comparison revealed higher concentrations of ITIH4 (*p* < 0.0006; *q* < 0.0165), SPA3 (SPA3N) (*p* < 0.0002; *q* < 0.0120), and HPT (*p* < 0.0004; *q* < 0.0146) and lower concentrations of A1AT2 (*p* < 0.0014; *q* < 0.0282), A1AT5 (*p* < 0.0010; *q* < 0.0238), and A2MG (PZP) (*p* < 0.0002; *q* < 0.0120).

For five out of the six samples for the SAA1 protein in the control mice, either expression changes were not detectable, or the protein concentration was so low that the protein simply could not be detected because of individual variation in the protein’s levels under different conditions. Because the samples were analyzed in duplicate (two replicates), these data were reliable. We included these proteins in the figure because significant differences were identified between aggressive and depressed animals.

The MUP1 protein (major urinary protein 1) was removed from the analysis owing to high variation in experimental groups and to its major function. According to the STRING database (Table S2, Supplement), MUP1 is primarily responsible for binding pheromones that are released from drying urine of mice. These pheromones affect the sexual behavior of females.

To avoid losing potentially useful information, we also examined differentially expressed proteins with *p* < 0.05. We analyzed proteins with altered expression in the losers compared with the controls and found 47 differentially expressed proteins (Supplement, Table S3): two amyloids, four apolipoproteins, three inter-alpha trypsin inhibitors, eight serine peptidase inhibitors, four complement components, nine globulins, and fourteen others. In the winners compared with the controls, we found 13 proteins (*p* value) (Supplement, Table S4): one apolipoprotein, two serine peptidase inhibitors, two complement components, and eight others. In the comparison of winners vs. losers (Supplement, Table S5), 57 differentially expressed proteins (*p* and/or *q* values) were found: three amyloids, four apolipoproteins, three inter-alpha trypsin inhibitors, eleven serine peptidase inhibitors, nine complement components, three globular proteins, and twenty other proteins. These findings confirmed that the protein expression changes were strongly specific to the depressed mice in comparison with the chronically aggressive mice.

Thus, the proteomic analysis of the plasma samples showed that the expression of proteins in the blood plasma of the depressed mice specifically changed under the chronic social defeat stress that was accompanied by the development of the depression-like state. These are the amyloid proteins SAA1, SAA4, and SAMP and apolipoproteins APOA2, APOC3, and APOD. Changed levels of serine protease inhibitors and/or proteins associated with lipid metabolism, inflammation, or immune function were also found in this comparison: upregulation of the proteins ITIH4, SPA3, HTP (HP), and CO9 (C9) and downregulation of the proteins A1AT2, A1AT5, and A2MG.

## 4. Discussion

It has been well confirmed that, after 20 days of agonistic interactions, a psychosis-like state develops in the winners ([12,22], reviews) and depression-like states develop in the losers ([14], review). The aim of this work was to study blood plasma markers in these social groups of mice having different pathological states. Our analysis of the blood plasma samples by mass spectrometry allowed for us to identify 14 proteins whose amounts differed significantly between samples grouped according to the social experience in the daily agonistic interactions in mice: “aggressive mice, winners” and “depressed mice, losers” in comparison with each other and with the control group of mice. These proteins are the most relevant and represent amyloids, apolipoproteins, trypsin and serine peptidase inhibitors, complement components, and globulins.

Amyloidosis is known to be a rare disease that occurs when amyloid proteins accumulate in systems of organs such as the nervous system and digestive tract and can lead to disturbances of their functions. High levels of amyloid proteins are associated with Alzheimer’s disease, which is accompanied by progression of cognitive deficits [27]. Extreme fatigue and weakness and weight loss or gain are also symptoms of depression in Alzheimer’s patients, implying comorbidity of these disorders. Moreover, serum amyloid A (SAA) participates in many immunomodulatory mechanisms, and its level increases in many inflammatory diseases [28]. In addition, SAA proteins affect the transport and absorption of cholesterol [29,30].

In the depressed mice compared with the control and aggressive mice, the levels of proteins were mostly higher (Figure 5 and Figure 6, Supplement, Tables S3–S5). From the overexpression of the amyloid proteins SAA4 and SAMP (encoded by genes *Saa4*, and *Apcs*) in the losers compared with the controls, we can deduce the formation of a predisposition to amyloidosis under chronic social stress in depressed mice to a greater extent as compared to the winners (which had a positive fighting experience), which did not differ from the control in these proteins’ levels.

Of note, the absence (“zero level”) of the SAA1 proteins (in five out of six samples) in blood plasma was found only in the control mice. According to GeneCards, diseases associated with the *Saa1* gene include serum amyloid A1 amyloidosis and amyloidosis. This protein also plays an important part in the metabolism of high-density lipoproteins (HDLs) and cholesterol homeostasis. High levels of this protein are linked with chronic inflammatory diseases, including atherosclerosis, rheumatoid arthritis, and Alzheimer’s disease, and are also a potential biomarker of certain tumors ([31], review). This is a very important fact in itself and shows that these proteins change their expression as a rule under the influence of experimental conditions to various degrees depending on a pathological state of mice, especially in depressed mice. Their presence in blood plasma can serve as a marker of an altered state and even progression of such a pathology.

Thus, the mostly elevated levels of amyloid proteins and apolipoproteins imply a psychopathogenic role of chronic social stress, which may cause a predisposition to disturbances in lipid metabolism. These data are consistent with those showing that overexpression of serum amyloid A1 induces depressive-like behavior in mice [32].

The most changed protein levels were documented for apolipoproteins ([33], review). The function of apolipoproteins is to bind lipids, including oil-soluble substances such as fats, cholesterol, and fat-soluble vitamins, to form lipoproteins. They transport lipids in the blood, cerebrospinal fluid, and lymph. Lipid components of lipoproteins are insoluble in water. Apolipoproteins and other biomolecules (such as phospholipids) can surround lipids, thus creating a lipoprotein particle that is itself water-soluble and can, therefore, be carried through body fluids (i.e., blood and lymph). Additionally, apolipoproteins interact with lipoprotein receptors and lipid transport proteins, thereby participating in lipoprotein uptake and clearance. They also serve as enzyme cofactors for specific enzymes involved in the metabolism of lipoproteins [33].

In the depressed mice, higher concentrations of proteins in the blood plasma were found for the apolipoproteins APOC3 and APOD, and lower levels were found for ADIPO proteins (apolipoprotein A-II, apolipoprotein C-III, and apolipoprotein D) encoded by genes *Apod*, and *Adipoq*, respectively. According to GeneCards, diseases associated with APOA2 proteins, encoded by genes *Apoa2*, include hypercholesterolemia and some types of amyloidosis. Among pathways related to APOA2, there are statin inhibition of cholesterol production as well as plasma lipoprotein assembly, remodeling, and clearance. Similarly to APOA2-related pathways, diseases associated with APOC3 include apolipoprotein C-III deficiency and coronary heart disease. GO annotations related to these genes include lipid and cholesterol binding [33].

Among the pathways related to APOD (apolipoprotein D), GO annotations related to this gene include transporter activity and cholesterol binding. *ApoD* gene expression is affected in several pathologies, such as HDL (high-density lipoprotein) familial deficiency and many others including central nervous system disorders and cancer.

Diseases associated with ADIPO proteins include adiponectin deficiency and fatty liver disease. Among the pathways related to ADIPO, there are AMP-activated protein kinase signaling and energy metabolism. The *Adipoq* gene is expressed in adipose tissue exclusively. The encoded protein circulates in plasma and is involved in metabolic and hormonal processes.

A comparison of plasma protein expression between patients with depression and healthy controls has revealed that differentially expressed proteins, including apolipoproteins such as the APOE, APOC4, and APOA5, lead to significant alterations of lipid and metabolic functions [6]. According to pathway analysis, the top statistically significant pathways were related to lipid transport, inflammation, and immunity. Network analysis of integration of differential expression of proteins and metabolites indicated that a disturbance of phospholipid metabolism may contribute to inflammation in the central nervous system [6]. It can be hypothesized that the diminished level of the ADIPO protein and the overexpression of the APOC3 protein in comparison with the controls and winners can be considered markers of a depression-like state.

We also found changes in the expression of serpin proteins, which are a widespread family of protease inhibitors that use conformational alterations to inhibit target enzymes [34,35,36]. Serpins play a central role in the control of many important proteolytic cascades. Serpins are conformationally labile, and many disease-associated serpins result in the misfolding or formation of pathogenic, inactive biopolymers. Approximately two-thirds of human serpins perform extracellular functions by inhibiting proteases in the bloodstream to modulate their activities [36]. Deficiencies of serpins have been implicated in dementia, thrombosis, chronic fatigue, essential hypertension, hypothyroidism, tumor progression, and other health problems [35,36,37,38,39,40,41]. Consequently, serpins join a growing number of structurally distinct biomolecules that can cause major degenerative diseases (such as prion disease, which is a type of proteopathy) or the amyloid proteins that form inclusions in Alzheimer’s disease [35,41].

In the depressed mice, changed levels of proteins associated with serpinopathies were registered in the plasma in comparison with the controls: elevated levels of the proteins ITIH4 and SPA3 (SPA3N, SPA3A, and SPA3G) and decreased levels of A1AT5 proteins and A2MG proteins, which are broad-spectrum protease inhibitors. These proteins are serine protease inhibitors and are encoded by genes *Itih4*, *Serpina3* (*Serpina3N*, *Serpina3A*, and *Serpina3G*), *Serpina1b*, and *Serpina1e*, respectively. There are many diseases associated with serpins and serpinopathies [41,42,43,44,45,46]. For example, the proteins A1AT2, A1AT5, and A2MG participate in alpha-1-antitrypsin deficiency in humans and are associated with liver disease and inflammation. GO annotations related to these genes include binding of an identical protein and protease.

The SPA3 protein, also called α-1-antichymotrypsin (AACT, ACT), is one of the inhibitors of serine proteases. It plays an important part in the anti-inflammatory and antiviral response. Elevated levels of SERPINA3 (encoded by the *SerpinA3* gene) have been observed in heart failure and neurological diseases. Many studies have linked the observed increased expression levels of the *SerpinA3* gene with its crucial role in several pathologies in various types of cancer, such as glioblastoma, breast cancer, colorectal cancer, and melanoma [35,47]. The brain disease most strongly associated with the *SerpinA3* gene is Alzheimer’s disease. It has been reported that the SERPINA3 protein may bind to β-amyloid proteins and, thus, makes up a component of amyloid deposits. Another brain disease linked to this protein’s upregulation is prion disease [34,35], which includes rapidly developing dementia, muscle stiffness, difficulty walking, and fatigue.

The analysis of plasma proteins in patients indicates that one of the candidate diagnostic biomarkers of depression may be the SERPIND1 protein, which is upregulated in depressed patients [48]. The data from our model of depression match these data, confirming the development of a depression-like state in mice under chronic social defeat stress [11,12,13].

Higher concentrations of the proteins alpha 1-antitrypsin and haptoglobin (HTP, HP) have been found in the plasma of depressed patients [49,50,51,52]. These findings are consistent with the hypothesis that MDD is accompanied by inflammatory shifts with higher levels of the APP protein (amyloid beta precursor protein). It has been reported that elevated amounts of the proteins haptoglobin (HP) and alpha-1-antichymotrypsin (SERPINA3) highly correlate with each other and with the severity of depression and negatively correlate with the thyroid-stimulating hormone response to thyrotropin [49]. These findings provide further evidence for an inflammatory response during depression.

Increased levels of the ITIH4 (inter-alpha-trypsin inhibitor heavy chain 4) protein are associated with such disorders as human immunodeficiency and virus infection. It has been shown that, in the blood plasma of patients with MDD in comparison with healthy controls, the ITIH4 protein concentration is significantly higher but not in patients with schizophrenia or bipolar disorder [5]. Moreover, the consistency of these results has been verified by proteomic analysis of postmortem dorsolateral prefrontal cortex samples of brain tissue from 16 MDD patients. These data suggest that plasma ITIH4 protein levels may be a potential diagnostic biomarker of MDD [52] with high specificity. In our depressed mice, the level of the ITIH4 protein was higher. Thus, these data confirm that this protein may be used as a peripheral biomarker of depression in humans and in our experiment—in depressed mice.

Complement component 9 (CO9, C9) is involved in the complement system, which is a part of the innate immune system [53]. It is thought that, in plasma, exosomal proteins CO9 together with other proteins are biomarkers helping to differentiate Alzheimer’ patients from healthy individuals [54,55,56]. Similarly to these findings, abnormal levels of complement component C9 and haptoglobin alpha chain (HPT) were found here in the depressed mice in comparison with the controls. These proteins are also associated with inflammation and immune function.

The level of the A2M protein (in mice: A2MG; alpha-2-macroglobulin) was lower in the depressed mice than in the chronically aggressive mice but not different from the controls. The α-macroglobulin (αM) family of proteins includes a broad-spectrum protease inhibitor that correlates with an elevated risk of Alzheimer’s disease [57,58,59] and is associated with all functions of α2-macroglobulin in the context of inflammation, immunity, and infections [59]. In mice the MUP1 proteins which is primarily responsible for binding pheromones, was identified also as a regulator for glucose and lipid metabolism [60].

Therefore, our study showed that, in mice, chronic social stress induces the development of a depression-like state that is accompanied by elevated levels of amyloid proteins and apolipoproteins in the blood plasma of depressed mice. Higher levels in the blood plasma of the depressed mice were documented for the amyloid proteins SAA1 and SAA4, for SAMP, and for apolipoproteins APOC3 and APOD, and lower levels were documented for ADIPO proteins. The proteins ITIH4, SPA3, A1AT5, A2MG, and CO9 (C9) are serine protease inhibitors and/or are associated with inflammation and innate immune function. As markers of a depression-like state, good candidates are the proteins SAA1, ITIH4, SPA3, and A1AT5, and are broad-spectrum protease inhibitors.

Right now, it is very difficult to interpret the changes in protein levels in the blood plasma of mice. In any case, however, these data indicate a psychopathogenic role of chronic social stress, which may create a predisposition to Alzheimer’s disease in humans during depression accompanied very often by immunodeficiency [15]. Our experimental approach may be useful for searches for peripheral biomarkers of depression in the hope to understand the comorbidity of depression with other diseases and to devise an appropriate treatment for the patients.

## 5. Conclusions

As described in many studies and reviews [61,62], there are many experimental approaches to study the mechanisms of psychoemotional disorders, in particular depression and anxiety, which are either a cause or a consequence of the development of other diseases. Our research confirms the existing ideas about the comorbidity of depression and anxiety and indicates that these pathological conditions are accompanied by many other diseases, creating a predisposition to Alzheimer’s disease, immune disorders, etc.

Our experimental data make it possible to understand the relation between chronic social stress, depression-like states, and immune function in male mice. The identification of specific proteins in blood plasma that show altered levels under chronic social defeat stress allows for us to study the molecular pathogenesis of depression and to identify potential biomarkers of comorbidities. If experimental studies reveal a correlation between proteins in blood plasma and changes in the brain, then in the future, this knowledge may help make a more accurate diagnosis, determine the stage of the disease, and predict consequences of this pathological condition. We think that this study opens up new possibilities for the design of novel therapeutic strategies against depression.

Limitation: There is much criticism of the use of animal models (reviews, [63,64]) to study pathological processes in depressed individuals. This is explained by the fact that there are many types of depression, which can be accompanied by various mental changes and somatic consequences developing concurrently with various psychoemotional aberrations and concomitant disorders and depending on heredity and life events. Naturally, researchers must take into account that currently available animal models are of limited use for these purposes because no one animal model fully reproduces this complex disease. However, it is obvious and inevitable that it is necessary to follow the path described in the excellent review “*From Laboratory to Life*” [61]: from experimental studies using adequate models of neurological and psychiatric diseases to the treatment of patients.

## Figures and Tables

**Table 1:** Weight of mice before and after 20 days of social conflicts.

	Losers	Winners	
Before	27.4 ± 0.42	27.8 ± 0.33	ns
After	28.9 ± 0.32	30.2 ± 0.39	*p* < 0.05
	*p* < 0.001	*p* < 0.001	

ns: not significant.

## Data Availability

All relevant data are available in the Supplementary Materials. Raw data are available from the authors upon request.

## Supplementary Materials

The following supporting information can be downloaded at: https://www.mdpi.com/article/10.3390/biomedicines12071485/s1, Supplementary Table S1: All proteins that were identified by LC–MS/MS in blood plasma of male mice; Supplementary Table S2: 15 Proteins of interest associated with diseases according to the STRING database for *Mus musculus*; Supplementary Table S3: The comparison of protein levels in blood plasma of mice (Controls vs. Losers, depressed mice); Supplementary Table S4: The comparison of protein levels in blood plasma of mice (Controls vs. Winners, aggressive mice); Supplementary Table S5: The comparison of protein levels in blood plasma of mice (Winners vs. Losers).

## References

[1] Duan J., Xie P. (2020). The potential for metabolomics in the study and treatment of major depressive disorder and related conditions. Expert Rev. Proteom..

[2] Woods A.G., Iosifescu D.V., Darie C.C. (2014). Biomarkers in major depressive disorder: The role of mass spectrometry. Adv. Exp. Med. Biol..

[3] Wu D., Peng Y., Zhou J., Yang Y.T., Rao C.L., Bai S.J., Zhou X.Y., Chen J., Liao L., Liang Z.H. (2015). Identification and validation of argininosuccinate synthase as a candidate urinary biomarker for major depressive disorder. Clin. Chim. Acta.

[4] Ren J., Zhao G., Sun X., Liu H., Jiang P., Chen J., Wu Z., Peng D., Fang Y., Zhang C. (2017). Identification of plasma biomarkers for distinguishing bipolar depression from major depressive disorder by iTRAQ-coupled LC-MS/MS and bioinformatics analysis. Psychoneuroendocrinology.

[5] Preece R.L., Han S.Y.S., Bahn S. (2018). Proteomic approaches to identify blood-based biomarkers for depression and bipolar disorders. Expert Rev. Proteom..

[6] Gui S.W., Liu Y.Y., Zhong X.G., Liu X., Zheng P., Pu J.C., Zhou J., Chen J.J., Zhao L.B., Liu L.X. (2018). Plasma disturbance of phospholipid metabolism in major depressive disorder by integration of proteomics and metabolomics. Neuropsychiatr. Dis. Treat..

[7] Wells D.A., Lennon S.R. (1989). Major depression and amyloidosis. Case Rep. Gen. Hosp. Psychiatry.

[8] Lin H.M., Gao X., Cooke C.E., Berg D., Labotka R., Faller D.V., Seal B., Hari P. (2017). Disease burden of systemic light-chain amyloidosis: A systematic literature review. Curr. Med. Res. Opin..

[9] Planton M., Raposo N., Albucher J.-F., Pariente J. (2017). Cerebral amyloid angiopathy-related cognitive impairment: The search for a specific neuropsychological pattern. Rev. Neurol..

[10] Elsworthy R.J., Aldred S. (2019). Depression in Alzheimer’s disease: An alternative role for selective serotonin reuptake inhibitors?. Rev. J. Alzheimers Dis..

[11] Kudryavtseva N.N., Bakshtanovskaya I.V., Koryakina L.A. (1991). Social model of depression in mice of C57BL/6J strain. Pharmacol. Biochem. Behav..

[12] Kudryavtseva N.N. (2000). Agonistic behavior: A model, experimental studies, and perspectives. Neurosci. Behav. Physiol..

[13] Galyamina A.G., Kovalenko I.L., Smagin D.A., Kudryavtseva N.N. (2017). Interaction of depression and anxiety in the development of mixed anxiety/depression disorder: Experimental studies of the mechanisms of comorbidity (review). Neurosci. Behav. Physiol..

[14] Kudryavtseva N.N. (2021). Development of mixed anxiety/depression-like state as a consequences of chronic anxiety: Review of experimental data. Current Topics in Behavioral Neurosciences.

[15] Keeney A.J., Hogg S. (1999). Behavioural consequences of repeated social defeat in the mouse: Preliminary evaluation of a potential animal model of depression. Behav. Pharmacol..

[16] Bartolomucci A., Leopardi R. (2009). Stress and depression: Preclinical research and clinical implications. PLoS ONE.

[17] Berton O., McClung C.A., Dileone R.J., Krishnan V., Renthal W., Russo S.J., Graham D., Tsankova N.M., Bolanos C.A., Rios M. (2006). Essential role of BDNF in the mesolimbic dopamine pathway in social defeat stress. Science.

[18] Golden S.A., Covington H.E., Berton O., Russo S.J. (2011). A standardized protocol for repeated social defeat stress in mice. Nat. Protoc..

[19] McKinney W.T., Bunney W.E. (1969). Animal model of depression. I. Review of evidence: Implications for research. Arch. Gen. Psychiatry.

[20] Kudryavtseva N.N., Shurlygina A.V., Galyamina A.G., Smagin D.A., Kovalenko I.L., Popova N.A., Nikolin V.P., Ilnitskaya S.I., Melnikova E.V., Trufakin V.A. (2019). Immunopathology of mixed anxiety/depression disorders: An experimental approach to studies of immunodeficiency states. Neurosci. Behav. Physiol..

[21] Kudryavtseva N.N. (1991). The sensory contact model for the study of aggressive and submissive behavior in male mice. Agress. Behav..

[22] Kudryavtseva N.N., Smagin D.A., Kovalenko I.L., Vishnivetskaya G.B. (2014). Repeated positive fighting experience in male inbred mice. Nat. Protoc..

[23] Kudryavtseva N.N. (2003). Use of the partition test in behavioral and pharmacological experiments. Neurosci. Behav. Physiol..

[24] Zavialova M., Kamaeva D., Kazieva L., Skvortsov V.S., Smirnova L. (2023). Some structural features of the peptide profile of myelin basic protein-hydrolyzing antibodies in schizophrenic patients. PeerJ.

[25] Carr S., Aebersold R., Baldwin M., Burlingame A., Clauser K., Nesvizhskii A. (2004). Working group on publication guidelines for peptide and protein identification data. The need for guidelines in publication of peptide and protein identification data: Working group on publication guidelines for peptide and protein Identification Data. Mol. Cell Proteom..

[26] Ravasi T., Suzuki H., Cannistraci C.V., Katayama S., Bajic V.B., Tan K., Akalin A., Schmeier S., Kanamori-Katayama M., Bertin N. (2010). An atlas of combinatorial transcriptional regulation in mouse and man. Cell.

[27] Ma C., Hong F., Yang S. (2022). Amyloidosis in Alzheimer’s disease: Pathogeny, etiology, and related therapeutic directions. Molecules.

[28] Fan Y., Zhang G., Vong C.T., Ye R.D., Kurouski D. (2019). Serum amyloid A and immunomodulation. Amyloid Diseases.

[29] Thomas M.J., Sorci-Thomas M.G. (2015). SAA: A link between cholesterol efflux capacity and inflammation?. J. Lipid Res..

[30] Ramasamy I. (2014). Recent advances in physiological lipoprotein metabolism. Clin. Chem. Lab. Med..

[31] Sack G.H. (2018). Sack Serum amyloid A—A review. Mol. Med..

[32] Jang W.Y., Lee B.R., Jeong J., Sung Y., Choi M., Song P., Kim H., Jang S., Kim H., Joo K.I. (2017). Overexpression of serum amyloid a 1 induces depressive-like behavior in mice. Brain Res..

[33] Dib I., Khalil A., Chouaib R., El-Makhour Y., Noureddine H. (2021). Apolipoprotein C-III and cardiovascular diseases: When genetics meet molecular pathologies. Mol. Biol. Rep..

[34] Sánchez-Navarro A., González-Soria I., Caldiño-Bohn R., Bobadilla N.A. (2021). An integrative view of serpins in health and disease: The contribution of SerpinA3. Am. J. Physiol. Cell. Physiol..

[35] Law R.H., Zhang Q., McGowan S., Buckle A.M., Silverman G.A., Wong W., Rosado C.J., Langendorf C.G., Pike R.N., Bird P.I. (2006). An overview of the serpin superfamily. Genome Biol..

[36] Davis R.L., Shrimpton A.E., Holohan P.D., Bradshaw C., Feiglin D., Collins G.H., Sonderegger P., Kinter J., Becker L.M., Lacbawan F. (1999). Familial dementia caused by polymerization of mutant neuroserpin. Nature.

[37] Xiao G., Liu Y.E., Gentz R., Sang Q.A., Ni J., Goldberg I.D., Shi Y.E. (1999). Suppression of breast cancer growth and metastasis by a serpin myoepithelium-derived serine proteinase inhibitor expressed in the mammary myoepithelial cells. Proc. Natl. Acad. Sci. USA.

[38] Silverman G.A., Bird P.I., Carrell R.W., Church F.C., Coughlin P.B., Gettins P.G., Irving J.A., Lomas D.A., Luke C.J., Moyer R.W. (2001). The serpins are an expanding superfamily of structurally similar but functionally diverse proteins. Evolution, mechanism of inhibition, novel functions, and a revised nomenclature. J. Biol. Chem..

[39] Lomas D.A., Carrell R.W. (2002). Serpinopathies and the conformational dementias. Nat. Rev. Genet..

[40] Lomas D.A. (2005). Molecular mousetraps, alpha1-antitrypsin deficiency and the serpinopathies. Clin. Med..

[41] Akbor M.M., Kurosawa N., Nakayama H., Nakatani A., Tomobe K., Chiba Y., Ueno M., Tanaka M., Nomura Y., Isobe M. (2021). Polymorphic SERPINA3 prolongs oligomeric state of amyloid beta. PLoS ONE.

[42] Bouton M.C., Geiger M., Sheffield W.P., Irving J.A., Lomas D.A., Song S., Satyanarayanan R.S., Zhang L., McFadden G., Lucas A.R. (2023). The under-appreciated world of the serpin family of serine proteinase inhibitors. EMBO. Mol. Med..

[43] Fra A., D’Acunto E., Laffranchi M., Miranda E. (2018). Cellular models for the serpinopathies. Methods Mol. Biol..

[44] Lucas A., Yaron J.R., Zhang L., Macaulay C., McFadden G. (2018). Serpins: Development for therapeutic applications. Methods Mol. Biol..

[45] D’Acunto E., Fra A., Visentin C., Manno M., Ricagno S., Galliciotti G., Miranda E. (2021). Neuroserpin: Structure, function, physiology and pathology. Cell. Mol. Life Sci..

[46] de Mezer M., Rogaliński J., Przewoźny S., Chojnicki M., Niepolski L., Sobieska M., Przystańska A. (2023). SERPINA3: Stimulator or inhibitor of pathological changes. Biomedicines.

[47] Wang Q., Yu C., Shi S., Su X., Zhang J., Ding Y., Sun Y., Liu M., Li C., Zhao X. (2019). An analysis of plasma reveals proteins in the acute phase response pathway to be candidate diagnostic biomarkers for depression. Psychiatry Res..

[48] Joyce P.R., Hawes C.R., Mulder R.T., Sellman J.D., Wilson D.A., Boswell D.R. (1992). Elevated levels of acute phase plasma proteins in major depression. Biol. Psychiatry.

[49] Maes M., Scharpe S., Bosmans E., Vandewoude M., Suy E., Uyttenbroeck W., Cooreman W., Vandervorst C., Raus J. (1992). Disturbances in acute phase plasma proteins during melancholia: Additional evidence for the presence of an inflammatory process during that illness. Prog. Neuropsychopharmacol. Biol. Psychiatry.

[50] Maes M., Delange J., Ranjan R., Meltzer H.Y., Desnyder R., Cooremans W., Scharpé S. (1997). Acute phase proteins in schizophrenia, mania and major depression: Modulation by psychotropic drugs. Psychiatry Res..

[51] Maes M., Scharpé S., Van Grootel L., Uyttenbroeck W., Cooreman W., Cosyns P., Suy E. (1992). Higher alpha 1-antitrypsin, haptoglobin, ceruloplasmin and lower retinol binding protein plasma levels during depression: Further evidence for the existence of an inflammatory response during that illness. J. Affect. Disord..

[52] Shi Y., Song R., Wang L., Qi Y., Zhang H., Zhu J., Zhang X., Tang X., Zhan Q., Zhao Y. (2020). Identifying plasma biomarkers with high specificity for major depressive disorder: A multi-level proteomics study. J. Affect. Disord..

[53] Lint T.F., Zeitz H.J., Gewurz H. (1980). Inherited deficiency of the ninth component of complement in man. J. Immunol..

[54] Goetzl E.J., Schwartz J.B., Abner E.L., Jicha G.A., Kapogiannis D. (2018). High complement levels in astrocyte-derived exosomes of Alzheimer disease. Ann. Neurol..

[55] Winston C.N., Goetzl E.J., Schwartz J.B., Elahi F.M., Rissman R.A. (2019). Complement protein levels in plasma astrocyte-derived exosomes are abnormal in conversion from mild cognitive impairment to Alzheimer’s disease dementia. Alzheimers Dement..

[56] Cai H., Pang Y., Wang Q., Qin W., Wei C., Li Y., Li T., Li F., Wang Q., Li Y. (2022). Proteomic profiling of circulating plasma exosomes reveals novel biomarkers of Alzheimer’s disease. Alzheimers Res. Ther..

[57] Kovacs D.M. (2000). Alpha2-macroglobulin in late-onset Alzheimer’s disease. Exp. Gerontol..

[58] Kovacs D.M., Blacker D., Wilcox M.A., Laird N.M., Rodes L., Horvath S.M., Go R.C., Perry R., Watson B., Bassett S.S. (1998). Alpha-2 macroglobulin is genetically associated with Alzheimer disease. Nat. Genet..

[59] Vandooren J., Itoh Y. (2021). Alpha-2-Macroglobulin in inflammation, immunity and infections. Front. Immunol..

[60] Zhou Y., Jiang L., Rui L. (2009). Identification of MUP1 as a regulator for glucose and lipid metabolism in mice. J. Biol. Chem..

[61] Tanaka M., Vécsei L. (2024). From Lab to Life: Exploring Cutting-Edge Models for Neurological and Psychiatric Disorders. Biomedicines.

[62] Tanaka M., Chen C. (2023). Towards a mechanistic understanding of depression, anxiety, and their comorbidity: Perspectives from cognitive neuroscience. Front. Behav. Neurosci..

[63] Czéh B., Fuchs E., Wiborg O., Simon M. (2016). Animal models of major depression and their clinical implications. Prog. Neuropsychopharmacol. Biol. Psychiatry.

[64] Hao Y., Ge H., Sun M., Gao Y. (2019). Selecting an appropriate animal model of depression. Int. J. Mol. Sci..

